# Psychological Sleep Interventions for Migraine and Tension-Type Headache: A Systematic Review and Meta-Analysis

**DOI:** 10.1038/s41598-019-42785-8

**Published:** 2019-04-23

**Authors:** Daniel P. Sullivan, Paul R. Martin, Mark J. Boschen

**Affiliations:** 1 School of Applied Psychology and Menzies Health Institute Queensland, Griffith University, Mount Gravatt, Australia; 2 Research School of Psychology, The Australian National University, Canberra, Australia

**Keywords:** Psychiatric disorders, Migraine

## Abstract

Disordered sleep, poor sleep quality, and insufficient or excessive sleep duration are known triggers of primary and secondary headaches. Given this, it is plausible that improving sleep will subsequently reduce headache activity. We report a systematic review of the literature, examining studies utilising psychological sleep interventions for the treatment of migraine and tension-type headache. PubMed, EMBASE, CINAHL, PsycINFO, and Cochrane Central were searched, using terms pertaining to psychological sleep interventions and headaches. Meta-analysis was performed for two outcome measures; headache frequency, and headache intensity. 103 studies were retrieved, of which 55 were duplicates. After completing reviews, three studies were retained. An additional eligible study was published after the initial search, and was found via monthly update searches, resulting in a total of four included studies. The effects of psychological sleep interventions (and in one study, combined with drug therapy) significantly reduced headache frequency and headache intensity. Three studies improved various sleep outcomes such as duration, efficiency, and excessive sleepiness. Psychological sleep interventions improve headache frequency and sleep, however there is conflicting evidence for the effect on headache intensity between studies. Limitations include the small number of studies conducted to date. Despite this, the notable improvements in headaches and sleep achieved after psychological sleep interventions indicates further research on this promising topic is warranted.

## Introduction

The medical and psychological literature demonstrates a convincing link between sleep problems and primary headaches^1^. Primary headaches are neurological disorders of head pain not directly attributable to another medical condition, the most common primary headaches are migraine and tension-type headache (TTH). Numerous sleep factors have been implicated in migraine and tension-type headaches, including sleep duration, sleep quality, obstructive sleep apnoea risk, and circadian rhythm misalignment^2^.

A recent meta-analysis examining the perceived triggers of migraine and tension-type headache (TTH) sufferers, found sleep (a broad category including triggers such as a change in sleep pattern, fatigue etc.) was the second most commonly reported headache trigger^3^. This is perhaps unsurprising, given all chronic migraine sufferers in a previous study by Calhoun, *et al*.^4^ reported problems with their sleep, and endorsed a high degree of maladaptive sleep behaviours.

Sleep problems and headache possibly co-occur as a result of the dysregulation of shared brain regions, i.e., issues with sleep trigger headaches, and headaches can lead to poor sleep^5^. The trigeminal nucleus caudalis (TNC) is the main area of the brainstem responsible for the sensation of head pain, which transmits to the somatosensory cortex via the ventral posteriomedial thalamus^5^. The experience of pain includes both a sensory component and an affective (emotional) component; the TNC innervates the limbic cortex, which has been implicated in emotional response to pain^5,6^. Additionally, the hypothalamus has been implicated in sleep-related headaches, as a controller of sleep and circadian rhythms^5^. Rapid eye movement sleep (REM) is regulated by so-called REM-on and REM-off cells in the brain^7^. The ventrolateral periaqueductal gray (vPAG) is one such region responsible for switching off REM sleep, and is supplied by orexinergic inputs from the lateral hypothalamus^5,7^. When stimulated with orexin, the vPAG has an inhibitory effect on nociception in the TNC^8^. Indeed, with the hypothalamus’ responsibility for sleep-wake rhythms, dysregulated sleep in general may disrupt hypothalamic signalling to the vPAG, potentially explaining the link between various issues with sleep dysregulation and headache activity.

Animal models show that when deprived of rapid eye movement sleep (REM), rats experience greater pain sensitivity^9^, and humans with insomnia spend less time in REM, and have REM that is more fragmented^10^. In human subjects, sleep disturbance has been demonstrated to worsen pain symptomatology^11^, as well as decreasing the effectiveness of central pain inhibitory processes^12^. Roehrs and Roth^13^ have reviewed the interactions between sleep and pain. When examining sleep and headaches prospectively, Houle, *et al*.^14^ found that two days of stress or inadequate sleep were associated with higher incidence of headaches, and conversely, two days of lowered stress and adequate sleep acted as a protective factor. Logically if dysregulated or disrupted sleep leads to headaches, improving sleep should reduce headache frequency and/or intensity. And whilst sedative/hypnotic medications play a role in the management of certain sleep problems, where the aetiology of the issue is psychophysiological, such as insomnia, psycho-behavioural interventions are the initial treatment of choice^15^.

Both behavioural and cognitive strategies exist for treating insomnia and poor sleep habits; when used together these are referred to as Cognitive Behaviour Therapy for Insomnia (CBT-i). Behavioural sleep modification strategies include sleep restriction/bed restriction, stimulus control, and sleep hygiene^16^. By restricting the opportunity for sleep to the average estimate of actual sleep duration, a greater sleep pressure (the drive to sleep) is accumulated, and consequently, sleep latency is reduced and total sleep time and sleep efficiency is increased^17^. Stimulus control aims to break the association between the sleeping environment and wakefulness that develops with insomnia and poor sleep habits. Stimulus control requires the patient to only go to bed when sleepy, and to get out of bed after being unable to sleep for approximately 20 minutes. Napping is also cautioned against, as this may reduce sleep pressure, making sleep during the main rest period more difficult^16,18^. Sleep hygiene encompasses optimal lifestyle and environmental factors for sleep. Lifestyle factors include avoiding nocturnal alcohol consumption due to its fragmenting effects on sleep, and avoiding stimulants such as nicotine and caffeine prior to bed^16^. Keeping excessive light and noise out of the bedroom are examples of environmental factors which are targeted in sleep hygiene education^16^.

CBT-i is indicated as a first-line therapy for treating insomnia^15^, and has been demonstrated to increase both REM and non-REM sleep^19^. Given the implication of sleep in pain, correcting poorly regulated sleep with psychological interventions may reduce headache frequency and/or intensity. There are no studies exploring the neurophysiology of how psychological sleep interventions may improve headaches. Evidence exists, however, where neurophysiological changes associated with behavioural and cognitive sleep interventions map onto the brain regions known to be shared between pain and sleep (see Brennan and Charles^5^). Lee, *et al*.^20^ studied subcortical resting state functional connectivity in insomnia patients before and after CBT-i. After treatment, they found a decrease in functional connectivity (FC) between the thalamus (a region with implications for both pain and sleep) and the parietal cortex^20^. The decreased FC between the thalamus and parietal cortex correlated inversely and significantly with sleep efficiency (i.e., decreased thalamo-parietal FC was associated with increased sleep efficiency)^20^. Another small study of four patients by Smith, *et al*.^21^ reported on regional cerebral blood flow (rCBF) during non-REM sleep, before and after behaviour therapy for insomnia. Single photon emission computed tomography (SPECT) was used, revealing a mean increase of 19% in blood flow to the thalamus^21^. This change, however, was statistically non-significant (p = 0.13), as two of the four patients did not have marked changes in thalamic rCBF^21^. Nonetheless, this finding is of interest when hypothesising the mechanisms of psychological sleep interventions and how they may lead to neural changes that affect headache activity.

Additionally, there is already evidence that dedicated psychological approaches for headache problems are effective. These programs include Cognitive Behaviour Therapy for headaches^22^, and more recently, a promising behavioural treatment approach, the Learning to Cope with Triggers (LCT) program^23^. It is possible that any benefits of sleep approaches may be partially derived from the overlap of techniques (e.g., relaxation training) also shared (and demonstrated as effective) by dedicated headache approaches.

Whilst there have been several excellent narrative reviews on this topic^24–26^, to date there has been no systematic review on the use of psychological sleep interventions for the treatment of headaches. As such, this study seeks to address that gap in the literature, and to synthesise new research on the topic published subsequent to prior reviews.

This review examined psychological sleep interventions to treat any headache type in all age groups. The primary outcomes were a reduction in headache frequency and/or intensity. Secondary outcomes assessed were improvements in sleep duration and/or quality. The timeframes over which the intervention and follow-up were performed were not specified. Permissible study designs included randomised controlled trials, or non-randomised trials.

## Results

### Study Selection

**Figure 1:**
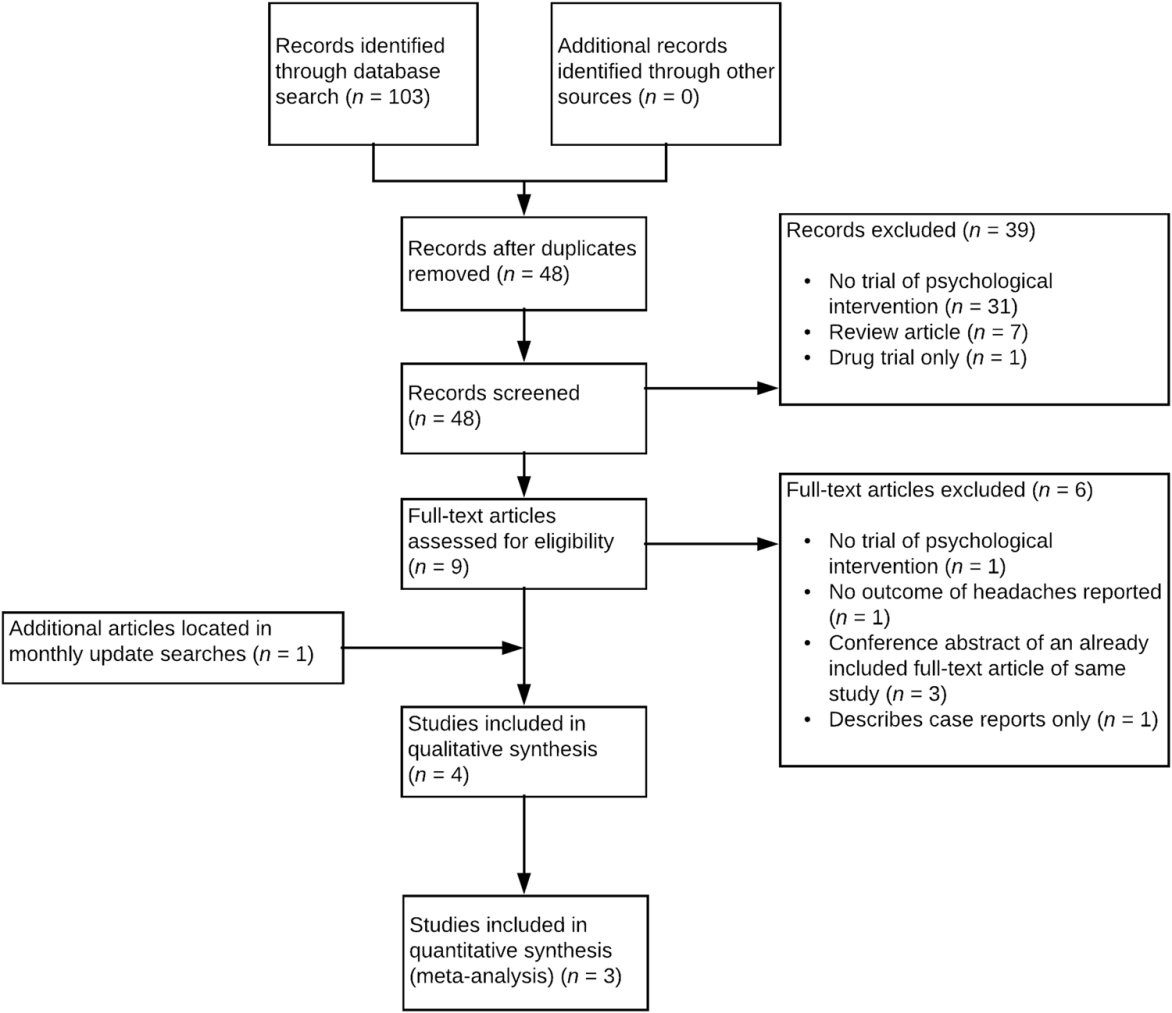
PRISMA flowchart demonstrating identification, inclusion, and exclusion of studies in the systematic review.

In total, 103 records were retrieved across all databases. After duplicate removal, title-abstract screening and full-text screening, only three studies met criteria for inclusion. Monthly update searches revealed an additional eligible study in the period after initial searches were completed. In total, four studies were included in the review. The PRISMA flow diagram is presented in Fig. 1.

### Study Characteristics

Characteristics of the three included studies are presented in Table 1.

**Table 1:** Characteristics of Included Studies.

Author (Year)	Country	Study Design	Sample Description	Intervention/Comparison	Relevant Outcomes & Follow-Up	Mean Age (Years)
Calhoun & Ford (2007)^29^	USA	Crossover pilot RCT	Adult females (*n* = 43) with chronic migraine. No primary sleep disorder.	1 session BSM/sham behavioural intervention^a^	Headache frequency, HA intensity, reversion to episodic migraine. Follow-up at 6, 12, 18 weeks post-treatment.	BSM: 33.5, Placebo: 35
Ruff *et al*. (2009)^28^	USA	Single-arm Pre-post	Adult male and female veterans (*n* = 126, Female = 7%) with blast induced mTBI and TTH, migraine or mixed HA.	1 session sleep hygiene and oral R_x_ Prazosin/no control group	HA frequency, HA intensity, sleepiness. Follow-up at 9 weeks, 6 months post-treatment.	Single arm intervention: 29.4
Smitherman *et al*. (2016)^30^	USA	Parallel-arm pilot RCT	Adult males and females (*n* = 32, Female = 90.3%) with chronic migraine and comorbid insomnia.	3 session CBT-i/sham behavioural intervention^a^	HA frequency, HA related disability, sleep efficiency, total sleep time, sleep quality, psychiatric Sx. Follow-up at 2, 6 weeks post-treatment.	CBT-i: 29.6, Placebo: 32.1
Law *et al*. (2018)^31^	USA	Single-arm Pre-post	Adolescent males and females (*n* = 21, Female = 81%) with chronic migraine or chronic TTH	6 session hybrid CBT-i^b^	HA frequency, HA intensity, pain related disability, insomnia Sx, sleep quality, sleep hygiene, total-sleep-time. Follow-up immediately post-treatment and 3-months post-treatment	Single-arm intervention: 15.5

*Abbreviations:* BSM, behavioural sleep modification; CBT-i, cognitive behaviour therapy for insomnia; HA, headache; mTBI, mild traumatic brain injury; RCT, randomised controlled trial; R_x_, prescribed medication; Sx, symptoms; TTH, tension-type headache.

^a^Sham behavioural intervention included consistent suppertime, performing acupressure, recording liquid consumption, range of motion exercises, consuming protein at breakfast. ^b^Hybrid CBT-i intervention blends CBT-i techniques with CBT techniques to target pain.

### Risk of Bias within Studies

**Figure 2:**
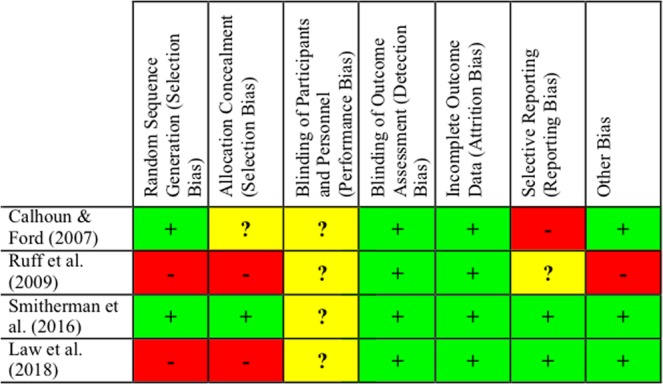
Risk of bias summary: Review authors’ judgements about each risk of bias item for each study included.

Studies included in the review were assessed for risk of bias using the Cochrane Risk of Bias tool^27^, categorising studies as high, low, or unclear risk of bias across seven domains. Risk of bias assessments are presented in Fig. 2.

### Meta-Analysis of Primary Outcomes

**Figure 3:**
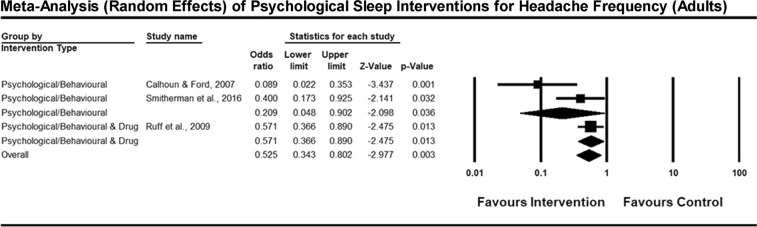
Forest plot of psychological sleep interventions for headache frequency in adults. *Note:* Exact P values were not provided by Ruff *et al*., therefore a two-tailed test with p = 0.049 was imputed for results reported as p < 0.05, and p = 0.051 for tests reported as non-significant. In the forest plot, square icons indicate individual studies. Diamond icons represent studies pooled together by intervention type, and the overall effect of all studies pooled together.

**Figure 4:**
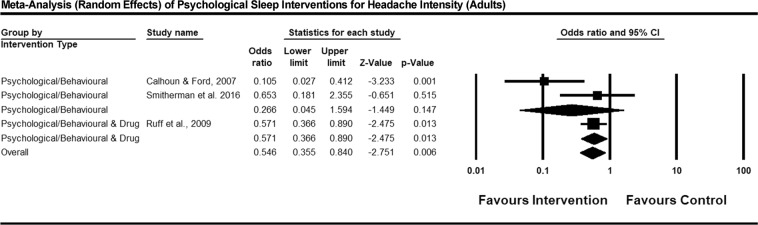
Forest plot of psychological sleep interventions for headache intensity in adults. *Note:* Exact P values were not provided by Ruff *et al*., therefore a two-tailed test with p = 0.049 was imputed for results reported as p < 0.05, and p = 0.051 for tests reported as non-significant. In the forest plot, square icons indicate individual studies. Diamond icons represent studies pooled together by intervention type, and the overall effect of all studies pooled together.

Figures 3 and 4 present forest plots for the effect of psychological sleep treatments on headache frequency, and intensity, respectively. Both figures report the results of a random-effects, generic inverse variance meta-analysis. In Ruff, *et al*.^28^, some participants either did not commence Prazosin therapy or discontinued before follow-up. In accordance with intention-to-treat analysis, all participants in the Ruff study were pooled together regardless of whether they were taking Prazosin at follow-up. Sleep outcomes were not subject to meta-analysis due to the highly varying domains of sleep that were measured in different studies.

### Synthesis of Results

#### Headache frequency and intensity

All included studies demonstrated decreased frequency of headaches in the intervention group/s. The group receiving active treatment in Calhoun and Ford^29^ reduced from an average of 24.2 to 17.4 headaches per 28 days. In veterans with migraine, TTH, and mixed features headaches, Ruff, *et al*.^28^ found a marked improvement in headache frequency in patients who completed sleep hygiene and continued to take Prazosin at the six-month follow-up. Baseline headache frequency in this group was 13.6 per month, and had reduced to 2.26 per month. In the small proportion (*n* = 8) of patients in Ruff, *et al*.^28^ not taking Prazosin at six-month follow-up (due to opting not to commence medication, or discontinuing medication), mean headache frequency per month reduced non-significantly from 7.19 to 6.89. Smitherman, *et al*.’s^30^ active group improved from 22.7 to 11.6 headache days per month. Law, *et al*.’s^31^ single-arm adolescent trial demonstrated a significant (*p* < 0.05) reduction in weekly headache frequency, reducing from 4.7 headache days per week at baseline to 2.7 days per week at final follow-up.

For the outcome of headache intensity, Calhoun and Ford^29^ found a significant reduction in the intervention group (*p* = 0.001), and the control group did not significantly reduce in headache intensity. In Ruff, *et al*.^28^ those taking Prazosin (and having had sleep hygiene training) experienced significant reductions in headache intensity between baseline and follow-up, however those not taking Prazosin at six-months did not experience significant reductions for this outcome. Both the intervention and control groups in Smitherman, *et al*.^30^ experienced significant reductions in headache intensity, and did not differ significantly at follow-up. The adolescents in Law, *et al*.^31^ did not significantly reduce their headache intensity (Pre: 5.2, Post: 4.6, *p* > 0.05), however pain related disability due to headache was significantly improved at follow-up compared to baseline (Pre: 32.7, Post: 21.19, *p* < 0.05)

#### Sleep quality, total sleep time, and sleep efficiency

The sleep variables measured and reported across studies were highly varied, making meta-analysis unsuitable. Calhoun and Ford^29^ collected baseline data using sleep items from the Medical Outcome Study (MOS)^32^. The sleep scale of the MOS asked self-report questions pertaining to domains including sleep latency, sleep duration, and how frequently subjects get sufficient sleep to feel rested. Sleep outcome data post-treatment was not reported by Calhoun and Ford^29^.

Ruff, *et al*.^28^ reported pre-post Epworth Sleepiness Scale (ESS) results as their sleep outcome measure. The ESS^33^ rates the patient’s subjective daytime sleepiness in a variety of situations (such as driving a vehicle, socialising etc.), with higher scores representing increasingly pathological sleepiness, indicative of a problem with the patient’s quantity or quality of sleep. Participants who were taking Prazosin had a significant and marked reduction in excessive daytime sleepiness, as did those who weren’t taking Prazosin at follow up. Those taking Prazosin at six-months reduced to an ESS of 4 (normal sleepiness), from a baseline of 16.2 (severe sleepiness); and those not on Prazosin (thus only equipped with sleep hygiene therapy) began with a mean ESS of 16 and this reduced to 10.9 (borderline normal/mild sleepiness).

Smitherman, *et al*.^30^ reported sleep duration and efficiency (as measured by actigraphy and sleep diaries), and sleep quality (measured by the Pittsburgh Sleep Quality Index^34^). They found an average improvement of 52.7 minutes’ sleep in the CBT-i group (*p* = 0.049, *η*^2^_*p*_ = 0.14) at follow-up. Whereas the sham group showed non-significant improvements of 5.9 minutes (*p* = 0.068) on average. The CBT-i group also demonstrated a statistically significant, 3.7% increase in sleep efficiency (*p* = 0.001, *η*^2^_*p*_ = 0.32), whereas the sleep efficiency of the sham group marginally worsened, decreasing by 0.3%.

The sleep outcomes of Law, *et al*.^31^ included insomnia symptoms (measured by Insomnia Severity Index^35^), sleep quality (measured by the Adolescent Sleep Wake Scale^36^) and sleep hygiene (measured by Adolescent Sleep Hygiene Scale^37^). Additionally, sleep latency, duration, wake-after-sleep-onset (WASO), and sleep efficiency were derived from electronic sleep diaries. Between baseline and final follow-up all sleep measures significantly improved (*p* < 0.05) as follows: insomnia symptoms (Pre: 16.9, Post: 9.3), sleep quality (Pre: 3.3, Post: 4.0), sleep hygiene (Pre: 4.5, Post: 4.9), sleep latency (Pre: 1 hour:15 mins, Post: 0:43), sleep duration (Pre: 7:36, Post: 8:25), WASO (Pre: 32 mins, Post: 10.6), sleep efficiency (Pre: 80.8%, Post: 90.8%).

### Between-Study Variability

Between-study variability was examined by assessing heterogeneity across studies for each intervention class (psychological/behavioural or psychological/behavioural & drug) for each outcome a meta-analysis was performed on. Cochrane’s Q was calculated for fixed-effects versions of the reported meta-analyses. For the psychological/behavioural and drug intervention class, there was only one study, therefore heterogeneity is not applicable. For the psychological/behavioural intervention types for the outcome of headache frequency, there was no evidence of statistically significant heterogeneity, *Q* (2) = 3.34, *p* = 0.07, *I*^2^ = 70.05%. Similarly, for the headache intensity meta-analysis, the test for heterogeneity was not statistically significant, *Q* (2) = 5.52, *p* = 0.06, *I*^2^ = 63.76%.

## Discussion

Based on four available studies, psychological/behavioural sleep treatments (and in one study, combined with Prazosin), overall, resulted in significant and substantial reductions in headache frequency, headache intensity, and headache related disability in adult and adolescent females and males. Three studies reported both pre-post outcome data for sleep parameters, finding that the interventions used improved sleep onset latency, WASO, sleep efficiency, sleep duration, sleep quality, and in one study, improved excessive daytime sleepiness. Given both sleep problems and headaches are highly prevalent and frequently comorbid, this treatment approach has potential for targeting two considerable public health problems at once.

Primarily, limitations of this review pertain to the small number of studies performed in this area. Given that this specialised approach to treating headaches is relatively new, only having first been demonstrated 10 years ago, it is somewhat expected that there are few studies in the literature. There are also limitations with the individual studies reviewed. In Smitherman, *et al*.^30^, the control group unexpectedly improved their headache intensity, weakening the effect in comparison to the intervention group. This is of interest, as the same sham intervention was used by Calhoun and Ford^29^ where the control group did not improve their measure of headache intensity. Smitherman, *et al*.^30^ note the differences between their study and the sham intervention in Calhoun and Ford^29^, specifically that Calhoun’s control intervention was of one session in duration, and Smitherman’s was three sessions. Given most of the sham instructions related to establishing regular eating and drinking habits, it is possible that this inadvertently improved headaches. This control intervention may have not have been truly inert because skipping meals is commonly reported as a headache trigger^30^. In Ruff, *et al*.^28^, the eight participants not taking Prazosin at follow-up (and therefore only receiving the benefit of sleep hygiene counselling at that time) did not significantly reduce headaches. Most participants in Ruff, *et al*.’s^28^ study were also diagnosed with Post Traumatic Stress Disorder (PTSD), and Prazosin is known to reduce nightmares, thus improving sleep quality^38^. Whilst the sleep hygiene intervention in the absence of Prazosin significantly improved sleep quality in the patients not taking the drug, it is possible that nightmares associated with PTSD lead to sleep problems of a severity where a low-level sleep hygiene intervention does not provide a magnitude of effect large enough to reduce headache activity. Law, *et al*.’s^31^ sample consisted of adolescents, many of whom were also taking medications for headache prophylaxis, such as anticonvulsants, tricyclic antidepressants, and melatonin. Thus the positive results may have been somewhat confounded by treatment-as-usual alongside the trialled sleep intervention.

Additionally, there were issues with reported statistics in studies, as we needed to impute exact *p* values for Ruff *et al*.^28^, and Calhoun and Ford^29^ did not provide follow-up outcome data for sleep variables or standard deviations for headache outcomes. When examining the study designs of included trials, half the studies included in this review were single-arm intervention studies without a control group, and most studies had relatively small sample sizes, degrading their statistical power. Thus, while the four studies have demonstrated positive results, there is a need to replicate their findings with high quality RCTs that have larger sample sizes and a truly inert placebo behavioural intervention.

There is also a need to examine in which groups of the population these findings are generalizable to, and where future focus should be in trialling psychological sleep interventions for headaches. For example, of the four studies reviewed, three studies utilised samples that consisted of very high proportions of female participants (Calhoun and Ford: 100%, Smitherman, *et al*.: 90.3%, Law, *et al*.: 81%). Thus, these promising interventions need further trials with male headache sufferers. Likewise, three of the four studies were conducted on adults only, with one study trialling a sleep intervention in adolescents. The efficacy of Law, *et al*.’s pilot RCT is promising, however with a mean age of 15.5(SD: 1.6) years, we have no evidence yet for the use of these interventions in middle childhood. Furthermore, the only two RCTs were limited to subjects with migraine headache. It would be beneficial if psychological sleep interventions for chronic TTH could be trialled in an RCT. Finally, it would be of interest to conduct imaging studies, such as with functional magnetic resonance imaging (fMRI), to attempt to elucidate potential neural correlates of these interventions in headache populations, and how the interventions may act to improve sleep and headache activity.

In conclusion, psychological/behavioural sleep treatments are still a relatively new approach for treating headaches, with trials having only been first described in the literature a decade ago. Only three studies have been performed to date using exclusively psychological/behavioural techniques, and one study combining behavioural techniques with medication in veterans. Our analysis of those studies in this review showed them to be effective for reducing headache frequency and intensity, and improving sleep parameters such as sleep quality and sleep time. The positive results from these trials provide a foundation for future research, which should address sample size and statistical power issues, as well as further trialling in males and children, and elucidating the neurophysiological mechanisms of the interventions by conducting imaging studies.

## Method

### Protocol and Registration

The review and protocol was not registered as this is not a PRISMA requirement. For transparency, however, the protocol was presented at a national behavioural medicine conference. The study protocol was amended after the initial search was followed from screening to final inclusion. The initial research question focussed on migraine and tension-type headache in adults only, and required interventions to be compared to a placebo/sham condition or other active treatment. However, upon including only two studies from the literature, the criteria were expanded to allow other headache types, participant ages, and trials without control groups.

### Eligibility Criteria

Studies were included based on having performed a psychological or behavioural intervention targeting sleep problems as a treatment for headaches. The specific psychotherapeutic techniques deemed eligible were not defined, to allow for any technique with therapeutic merit to be used. Included studies were not limited to Randomised Controlled Trials (RCTs); pseudo-randomised, non-randomised, and single-group studies were also permissible. The inclusion of study designs other than RCTs is reflective of the relative infancy of the approach of using psychological sleep interventions for headaches; as such, the possibility had to be considered that there may have been few or no RCTs. There was no limit on the age of studies to be included, as a review on this topic had not been previously conducted. English language publications only were considered for inclusion. Studies were permitted to enrol participants of any age or headache type. A study must have used a valid measure or method for distinguishing headache types as a requirement to be included, which was judged by the reviewer upon full-text review.

### Information Sources

The databases used to search for studies for this review were: PubMed, EMBASE, CINAHL (via EBSCOhost), PsycINFO (via Ovid), and Cochrane Central. Databases were searched from their inception to present, with searches conducted between December 2016 and February 2017. Follow up searches of PubMed were conducted in May 2017 to check for eligible studies using old diagnostic terminology. Monthly automatic update searches via Ovid occurred indefinitely during the preparation of this review to locate new eligible studies.

### Search Strategy

Mateen, *et al*.^39^ compared title-only screening against title-abstract when conducting a medical systematic review. They found that both methods returned the same articles for inclusion in the review, but that title-only screening significantly reduced screening burden on reviewers. As such, title-only searches formed the search strategy for this review, in light of the findings of Mateen, *et al*.^39^ suggesting relevant articles should be locatable based on their titles only, with no disadvantage compared to title-abstract searching. The PubMed search was updated and re-checked in May 2017 for studies that may have used the old diagnostic label of ‘tension headache’ rather than ‘tension-type headache’. No additional relevant studies were located, and so the original search criteria were retained. The following example shows the search method used for the PubMed database.

(sleep[ti] OR insomnia[ti]) AND (psych*[ti] OR cogniti*[ti] OR behav*[ti] OR mindful*[ti] OR therap*[ti] OR treat*[ti] OR intervention[ti] OR modif*[ti]) AND (headache*[ti] OR migraine*[ti] OR tension-type[ti])

### Data Collection Process and Data Items

We extracted reported statistics from included studies to facilitate narrative and quantitative synthesis. Where necessary, effect sizes were converted for inclusion in meta-analysis.

Variables from which data was sought included headache variables, specifically, the measure/method of headache diagnosis, the headache diagnosis given to participants, baseline and post-treatment frequency and/or severity of headache. Sleep variables sought were baseline and post-treatment sleep latency, sleep efficiency, total sleep time (TST) and sleep quality. The study details sought were descriptions of the sample and its size, a measure of treatment effect size, the design of the study, the description of the psychotherapeutic intervention used, attrition, and follow-up period.

### Risk of Bias in Individual Studies and Between-Study Heterogeneity

Individual studies were assessed for bias using the Cochrane risk of bias tool^27^. The tool assesses seven possible domains of bias within individual studies, including random sequence generation, allocation concealment, blinding, incomplete outcome data, selective reporting, and any other suspected bias. Outcome level assessment of across study heterogeneity was examined using the Cochrane Q and I^2^ metrics.

### Summary Measures, Synthesis of Results, and Additional Analyses

All reported statistics were converted to odds ratios for the purposes of quantitative pooling. Statistical analysis and meta-analytic synthesis of results was performed using Comprehensive Meta-Analysis^40^.

Sub-group analyses for headache-types were initially planned, however the small pool of included studies and headache types were not sufficient to perform such analyses.

## Data Availability

Findings of this systematic review are based on the data reported in articles previously published by Calhoun and Ford^29^, Ruff, *et al*.^28^, Smitherman, *et al*.^30^, and Law, *et al*.^31^.
